# Anesthetic management of hepatectomy for the patient with Fontan circulation: a case series

**DOI:** 10.1186/s40981-022-00582-9

**Published:** 2022-12-09

**Authors:** Makoto Sumie, Nozomi Kameyama, Taiki Akasaka, Yuto Tanaka, Taichi Ando, Tadashi Kandabashi, Yuji Karashima, Ken Yamaura

**Affiliations:** 1 Department of Anesthesiology and Critical Care Medicine, Kyushu University Hospital, 3-1-1 Maidashi, Higashi-Ku, Fukuoka, 812-8582 Japan; 2 Department of Anesthesia, Takagi Hospital, 141-11 Sakemi, Okawa-Shi, Fukuoka, 831-0016 Japan; 3 Department of Anesthesia, Saiseikai Fukuoka General Hospital, 1-3-46 Tenjin, Chuo-Ku, Fukuoka, 810-0001 Japan; 4 Medical Information Center, Kyushu University Hospital, 3-1-1 Maidashi, Higashi-Ku, Fukuoka, 812-8582 Japan; 5 Operating Rooms, Kyushu University Hospital, 3-1-1 Maidashi, Higashi-Ku, Fukuoka, 812-8582 Japan

**Keywords:** Fontan circulation, Hepatocellular carcinoma, Hepatectomy, Laparoscopic

## Abstract

**Background:**

Hepatectomy for patients with Fontan circulation consists of high central venous pressure and low pulmonary vascular resistance, and is challenging for physicians.

**Case presentation:**

We performed anesthetic management for hepatectomy in three patients with Fontan circulation. Massive bleeding and transfusion as well as careful management were needed. Open abdominal surgery had to be conducted instead of laparoscopic surgery for controlling bleeding in one case. We successfully performed general anesthesia using nitric oxide and inotropes while monitoring arterial pressure and central venous pressure in all the cases.

**Conclusions:**

To maintain Fontan circulation during hepatectomy, it is important to manage central venous pressure and ensure appropriate circulatory volume.

## Background

Fontan circulation is an iatrogenic and definitive palliative condition for patients with single-ventricle physiology, wherein venous blood returns to the pulmonary circulation without passing through the right ventricle. The central venous pressure (CVP) drives the pressure of the pulmonary circulation, which must have low pulmonary vascular resistance (PVR). With the recent improvement in survival rate after Fontan surgery, hepatocellular carcinoma is one of the most emerging complications. Impaired hepatic oxygen delivery and increased CVP appear to pathophysiological mechanism of developing hepatocellular carcinoma in Fontan physiology [1]. Several case reports of hepatic resection in patients with Fontan circulation have been reported earlier, but information on its perioperative anesthetic management is sparse [2–5, 7–10].

## Case report

Patient’s profiles, preoperative data, and medication are summarized in Table 1. All patients were classified as Child–Pugh classification A.

**Table 1:** Preoperative characteristics of the three cases

	**Case 1**	**Case 2**	**Case 3**
Age (years)/Sex	21/F	28/F	43/M
Tumor size and location	19 mm in segment 2 36 mm in segment 4	80 mm in segment 6 30 mm in segment 7	23 mm in segment 3
SpO_2_ (%)	98	94	92
SVEF (%)	54	51	72
CVP (mmHg) ^a^	12	20	18
mPAP (mmHg) ^a^	11	17	14
PVR (wood units) ^a^	1.3	1.6	1.5
ScvO_2_ (%)^a^	68	83	66
CI (L/min/m^2^) ^a^	2.4	3.6	2.1
Hb (g/dL)	12.2	13.0	13.0
Platelet count (10^3^/µL)	184	174	139
PT-INR	1.28	1.03	1.23
APTT (s)	58	32.7	59
Child–Pugh classification	A	A	A
Antiplatelet drug	+	+	-
Anticoagulant drug	+	-	+
Heparin bridging	+	-	+

^a^Values from cardiac catheterization are within one month prior to surgery

*APTT* Activated partial thromboplastin time, *CI* Cardiac index, *CVP* Central venous pressure, *F* Female, *Hb* Hemoglobin, *INR* International normalized ration of partial thrombin time, M male, mPAP mean pulmonary artery pressure, PVR pulmonary vascular resistance, SpO_2_ oxygen saturation, ScvO_2_ central venous blood oxygen saturation, SVEF systemic ventricular ejection fraction

### Case 1:

Laparoscopic segmentectomy was planned in a 21-year-old woman (height, 157 cm; weight, 48 kg) with a history of Fontan surgery (Extracardiac Fontan) at the age of three for a right single ventricle and pulmonary atresia. She was on aspirin and warfarin, and was shifted to continuous infusion of heparin 500 units/h 7 days before surgery. Her blood pressure (BP) was 113/55 mmHg and heart rate (HR) was 70 beats/min. Laboratory data were within normal range except coagulation parameters. Details of hemodynamic parameters and abnormal laboratory data are described in Table 1.

General anesthesia was successfully induced with fentanyl 6 µg/kg, midazolam 0.08 mg/kg, rocuronium 1 mg/kg, followed by maintaining with sevoflurane 0.5–1.0 minimum alveolar concentration (MAC) and remifentanil 0.25–0.5 µg/kg/min. In addition to standard monitoring, invasive arterial blood pressure (ABP) via radial artery, CVP, central venous blood oxygen saturation (ScvO_2_), and stroke volume variation (SVV) were observed with the use of the FloTrac™/-HemoSphere™ system (Edwards Lifesciences, Irvine, CA, USA) and transesophageal echocardiography (TEE). We maintained a train-of-four (TOF) count of 1 or less as the levels of neuromuscular blockade. Dobutamine 2–4 µg/kg/min and nitric oxide 5 ppm were continuously administered through infusion and inhalation, respectively. We maintained pressure control ventilation (PCV), with pressure at 13–18 cmH_2_O, and positive end expiratory pressure (PEEP) at 5 cmH_2_O. Partial pressure of arterial carbon dioxide (PaCO_2_) was maintained at 35–45 mmHg. Pneumoperitoneal pressure was set at 8 mmHg. We maintained CVP at 10–15 mmHg, and hemoglobin (Hb) level over 12 g/dL to transfuse 3,380 mL of red cell concentrate (RCC) and 1,920 mL of fresh frozen plasma (FFP) with adequate urine output. ScvO_2_ was stable between 66 and 78% during surgery, and between 74 and 86% after extubation. SVV was remained between 5 and 13%. The intraoperative blood loss was 2,650 g. Rectus sheath block and intravenous patient-controlled analgesia (IVPCA) using fentanyl were performed for postoperative pain management. Extubation was performed smoothly about 30 min after the operation. Postoperative pain was less than 3 as Numerical Rating Scale (NRS) through second postoperative days.

### Case 2:

Open abdominal segmentectomy was planned for large hepatocellular carcinoma in a 28-year-old woman (height, 157 cm; weight, 45 kg) who underwent Fontan surgery (Lateral tunnel Fontan) at the age of four for corrected transposition of the great arteries, mitral atresia, and pulmonary atresia. She was on aspirin, which was stopped 7 days before surgery. Her BP was 103/61 mmHg and HR was 80 beats/min. Other hemodynamic and laboratory data are described in Table 1.

General anesthesia was induced with fentanyl 2 µg/kg, propofol 1.5 mg/kg, rocuronium 0.6 mg/kg and maintained with desflurane 0.8 MAC and remifentanil 0.2–0.3 µg/kg/min. Monitoring and neuromuscular blocking were performed similar to that in case 1. We adjusted PCV with PEEP to maintain PaCO_2_ of 35–45 mmHg. Continuous infusion of noradrenaline 0.02–0.04 µg/kg/min and inhalation of nitric oxide 5 ppm were performed for maintaining CVP at 15–20 mmHg. We transfused 560 mL of RCC and 720 mL of FFP to maintained a Hb level of 12 g/dL. ScvO_2_ was remained between 63 and 74% during surgery, and between 71 and 84% after extubation. SVV was remained between 4 and 12%. The hepatic segmentectomy was performed as scheduled and the intraoperative blood loss was 1,181 g. Transversus abdominis plane block and IVPCA were performed. Extubation was performed smoothly approximately 40 min after the operation. NRS was less than 4 through second postoperative days.

### Case 3:

Laparoscopic segmentectomy was planned in a 43-year-old man (height, 166 cm; weight, 58 kg) who underwent Fontan surgery (Atrio-pulmonary connection Fontan) at the age of three for tricuspid atresia. He was on warfarin, which was shifted to continuous infusion of heparin 500 units/h 5 days before surgery. His BP was 77/48 mmHg and HR was 74 beats/min. Laboratory data were within normal range except coagulation parameters. Details of hemodynamic parameters and abnormal laboratory data are described in Table 1.

General anesthesia was induced with fentanyl 2 µg/kg, propofol 1 mg/kg, rocuronium 0.6 mg/kg and maintained with desflurane 0.8 MAC and remifentanil 0.2–0.3 µg/kg/min. Monitoring, neuromuscular blocking, and adjustment of PCV to maintain PaCO_2_ were done as in the previous two cases. Dobutamine 3 µg/kg/min and noradrenaline 0.02–0.1 µg/kg/min were continuously infused. The hepatic segmentectomy was performed; however, open abdominal procedure was conducted instead of laparoscopic surgery due to massive bleeding. Although the bleeding was controlled rapidly, the amount of bleeding was 910 g. ABP decreased from 88/61 mmHg to 76/46 mmHg, CVP decreased from 17 to 14 mmHg, ScvO_2_ decreased slightly to less than 80%, and SVV remained at approximately 11%. TEE examination did not show obvious changes in diameter and wall motion of ventricle. Total intraoperative blood loss was 2,850 g. We transfused 1,960 mL of RCC and 2,520 mL of FFP. We maintained CVP at 13–20 mmHg, and Hb level over 12 g/dL during the operation. ScvO_2_ was stable between 77 and 86%. SVV was maintained between 7 and 13%. Extubation was performed smoothly about 16 min after the operation. IVPCA and intravenous acetaminophen were administered for postoperative pain. NRS was between 3 and 6 through second postoperative days.

## Discussion

In recent years, some case reports describe the anesthetic contrivance against hepatectomy for the patient with Fontan physiology. Maeda, et al. reported that continuous thoracic paravertebral block was effective for perioperative pain management and hemodynamic stability [2]. In our cases, case 1 and 2 were lower postoperative NRS than case 3 and obtained stable hemodynamic to conduct peripheral nerve brock. This result is consistent the effect of nerve block as Maeda’s report. Saito, et al. reported successful management of laparoscopic surgery using noninvasive monitoring system, such as a pulse contour cardiac output catheter [10]. We also conducted ABP, CVP, ScvO_2_, SVV, and TEE monitoring to assess Fontan circulation as a hemodynamic monitoring. However, to the best of our knowledge, there was no report of laparoscopic surgery which had to be converted open abdominal surgery for intraoperative massive bleeding as in case 3. We compare our cases each other and discuss the perioperative anesthetic management focused on CVP, intraoperative blood loss, and hemodynamic stability.

One concerns for hepatectomy with Fontan circulation is that high CVP might cause large amount of bleeding from the dissection surface while CVP is driving pressure of pulmonary circulation. Moreover, liver dysfunction and anticoagulation increase the risk of bleeding. We managed intraoperative CVP and Hb at preoperative levels to maintain Fontan circulation in fluid management and blood transfusion, as in previous case reports [3–5]. Ideal intraoperative CVP should be prioritized for Fontan circulation. However, some studies have suggested that the recommended CVP is less than 4 mmHg for hepatectomy without Fontan circulation, which is a conflicting management for patients with Fontan circulation [6]. The intraoperative blood loss in our cases ranged from 1,181 to 2,850 g, while other studies have reported an intraoperative blood loss of 100–4,100 g [2–5, 7–10]. The blood loss was not always massive even with high preoperative CVP and an open abdominal surgery, as in case 2. Although pneumoperitoneum could decrease blood loss by increasing the intra-abdominal pressure, pneumoperitoneal pressure was set modestly due to concerns about increasing the intrathoracic pressure in our patients. The tumor location, size, and ease of surgery might also affect the amount of bleeding. Laparoscopic surgery was administered according to the predictor of surgical difficulty such as extent of resection and location of tumor, even though Fontan physiology [11]. However, we should have preoperative discussions that involve cardiologists, surgeons, and anesthesiologists, and prepare the patients for possible conversion from laparoscopic to open surgery if the massive bleeding disturbs the surgical field.

We adjusted intraoperative inotropes when the BP was low, even though CVP was maintained. Although noradrenaline was administered to maintain the systemic vascular resistance (SVR) in cases 2 and 3, it should be administered carefully because high doses of noradrenaline increase PVR. Vasopressin may help in maintaining SVR without increasing PVR. We administered and adjusted dobutamine to support cardiac contraction in cases 1 and 3 after evaluating heart contractility.

In the present three cases, SVV and TEE monitoring besides CVP were conducted to evaluate circulating blood volume. Moreover, ScvO_2_ was conducted to evaluate Fontan circulation. The information from FloTrac™/-HemoSphere™ system and TEE may help in decision-making, because it is monitoring mixed venous blood oxygen saturation and CI from the Swan-Ganz catheter in the Fontan circulation is difficult. In cases 1 and 2, ScvO_2_ was higher after extubation than during surgery. This might suggest that spontaneous respiration is ideal for Fontan physiology. SVV was remained approximately 13% in our three cases, even though the massive bleeding as in case 3. After all, ABP and CVP might be the most sensitive and useful parameters to manage circulation volume during massive bleeding. It is unclear whether SVV estimates the circulation volume or not, because the algorithm of SVV does not include Fontan circulation. There are few reports of assessing fluid responsiveness using SVV in patients with Fontan circulation. Further studies are needed in this regard.

## Data Availability

Not applicable.

## Abbreviations

ABP: Arterial blood pressure
APTT: Activated partial thromboplastin time
BP: Blood pressure
CI: Cardiac index
CVP: Central venous pressure
EF: Ejection fraction
FALD: Fontan associated liver disease
FFP: Fresh frozen plasma
Hb: Hemoglobin
HCC: Hepatocellular carcinoma
HR: Heart rate
IVPCA: Intravenous patient-controlled analgesia
MAC: Minimum alveolar concentration
NRS: Numerical rating scale
PaCO_2_: Partial pressure of arterial carbon dioxide
PCV: Pressure control ventilation
PEEP: Positive end expiratory pressure
PT-INR: International normalized ration of partial thrombin time
PVR: Pulmonary vascular resistance
RCC: Red cell concentrate
ScvO_2_: Central venous blood oxygen saturation
SpO_2_: Oxygen saturation
SVV: Stroke volume variation
TOF: Train-of-four
TEE: Transesophageal echocardiography
TTE: Transthoracic echocardiography
